# Liver Disease Undernutrition Screening Tool Questionnaire Predicts Decompensation and Mortality in Cirrhotic Outpatients with Portal Hypertension

**DOI:** 10.3390/nu15173780

**Published:** 2023-08-29

**Authors:** Diego Casas-Deza, Vanesa Bernal-Monterde, Elena Betoré-Glaria, Ana Belén Julián-Gomara, Carmen Yagüe-Caballero, Alejandro Sanz-París, Eva María Fernández-Bonilla, Javier Fuentes-Olmo, Jose M. Arbones-Mainar

**Affiliations:** 1 Gastroenterology and Hepatology Department, Miguel Servet University Hospital, 50009 Zaragoza, Spain; vbernalm@gmail.com (V.B.-M.); elenab_ejea@hotmail.com (E.B.-G.); belen_zaj@hotmail.com (A.B.J.-G.); carmenyaguecaballero@gmail.com (C.Y.-C.); evaferbo@yahoo.es (E.M.F.-B.); fuentesolmo@gmail.com (J.F.-O.); 2 Instituto de Investigación Sanitaria de Aragón (IISA), 50009 Zaragoza, Spain; sanzparisalejandro@gmail.com (A.S.-P.); jmarbones.iacs@aragon.es (J.M.A.-M.); 3 Endocrinology and Nutrition Department, Miguel Servet University Hospital, 50009 Zaragoza, Spain; 4 Instituto Aragonés de Ciencias de la Salud (IACS), 50009 Zaragoza, Spain; 5 Centro de Investigación Biomédica en Red Fisiopatología de la Obesidad y Nutrición (CiberOBN), Instituto de Salud Carlos III, 28029 Madrid, Spain

**Keywords:** liver cirrhosis, LDUST, undernutrition, Child–Pugh, MELD

## Abstract

Background: Numerous scores are designed to predict outcomes of patients with liver cirrhosis. Our study aimed to evaluate the ability of the Liver Disease Undernutrition Screening Tool (LDUST) in predicting mortality and decompensation in outpatients with clinically significant portal hypertension (CSPH). We hypothesized that LDUST could help identify patients in need of nutritional supplementation and intervention. Methods: A prospective study of 57 CSPH patients (36.8% female, mean age: 63.5 ± 9.9 years) with a median follow-up of 41 months was conducted. Baseline liver function, nutrition, and sarcopenia were assessed, alongside LDUST. During follow-up, the occurrence of liver decompensation, hospital admission, need for emergency care, and mortality were evaluated. Results: A total of 56.1% of patients were Child A, and the most frequent etiology was alcohol (50.9%). Malnutrition risk according to LDUST raised mortality (HR: 25.96 (1.47–456.78)), decompensation (HR 9.78 (2.08–45.89)), and admission (HR 4.86 (1.09–21.61)) risks in multivariate Cox analysis. Combining LDUST with Child and MELD scores improved their decompensation prediction (0.936 vs. 0.811 and 0.866 vs. 0.700). Conclusions: The LDUST has a solid ability to predict complications in cirrhosis outpatients with CSPH, and its integration with Child and MELD models enhances their predictive power. LDUST implementation could identify individuals necessitating early nutritional support.

## 1. Introduction

Liver cirrhosis is the final and irreversible condition of many liver diseases [1]. It causes significant morbidity and mortality, particularly during patient decompensation, being the seventh leading cause of death in Europe. While established liver function scores like the Child–Pugh and MELD exhibit strong mortality correlations [2,3,4,5], their exclusive reliance on analytical data and cirrhosis-related complications entails inherent limitations. Likewise, more recent scoring systems such as CLIC-SOFA and CLIC-C have also shown a great accuracy in predicting short-term and long-term mortality in patients with acute-on-chronic liver failure and adverse outcomes associated with chronic liver disease (reviewed in Rashed et al. [6]). However, their calculation can be difficult due to the combination of many indicators [7].

Significantly, prevailing among patients with liver cirrhosis are concurrent conditions, including frailty, malnutrition, sarcopenia, and minimal hepatic encephalopathy, which have progressively gained prognostic relevance [8,9,10,11]. Nevertheless, the distinctive fluid retention dynamics in ascites and edema characteristic of liver cirrhosis curtail the applicability of general population assessment tools. To address this, specific evaluations for cirrhosis patients have emerged, such as the Royal Free Hospital-Nutrition Prioritizing Tool (RFH-NPT) and the Liver Disease Undernutrition Screening Tool (LDUST) [12,13], both endorsed by the European Society for the Study of the Liver (EASL) [14]. 

The LDUST [15], a self-administered six-question patient-reported outcomes (PROs) questionnaire, focuses on dietary intake, weight fluctuations, daily activity capacity, muscle/fat mass changes, and fluid retention. Patient responses are categorized as column A, B, or C, with two or more B or C answers indicating undernutrition risk. This straightforward method offers practical malnutrition risk assessment in clinical practice. However, its potential in predicting broader clinical outcomes within cirrhosis patients remains unexplored.

Hence, our study endeavors to fill this research gap by prospectively investigating the predictive capacity of LDUST scores in patients with liver cirrhosis and clinically significant portal hypertension (CSPH). We hypothesize that LDUST scores can provide insights into diverse clinical outcomes beyond malnutrition risk assessment. In doing so, we contribute to a more comprehensive understanding of cirrhosis prognosis and enhance our ability to tailor patient care effectively. 

## 2. Materials and Methods

### 2.1. Study Design and Population

This is a longitudinal, non-interventional, prospective cohort study carried out in the outpatient clinic of the hepatology unit at the Miguel Servet University Hospital, Zaragoza, Spain. 

All outpatients with cirrhosis and CSPH were consecutively recruited during April 2019. The diagnosis of liver cirrhosis was made using a combination of clinical, radiological, analytical, and histopathological criteria. The presence of CSPH was defined according to Baveno VII criteria as the presence of any of the following: (1) the presence of clinical manifestations arising from portal hypertension (namely, gastro-esophageal varices and ascites), (2) a transitional elastography value ≥ 25 kPa, (3) radiological findings diagnostic of portal hypertension such as portosystemic collateral vessels, (4) a portal pressure gradient ≥ 10 mmHg [16]. Compensated cirrhosis was defined by the absence of present complications of cirrhosis. The events that defined decompensation were overt ascites (or pleural effusion with increased serum ascites albumin gradient [>1.1 g/dL]), overt hepatic encephalopathy (West Haven grade ≥ II), and variceal bleeding.

The following exclusion criteria were applied: patients with an active diagnosis of malignant pathology, heart failure with NYHA grade ≥ 2, severe pulmonary hypertension, active infection, and/or use of enteral nutritional supplements were excluded. These exclusions were made due to the substantial impact these conditions have on the life expectancy of patients, potentially introducing bias to the results. Patients with severe psychiatric illness that could interfere with completing the questionnaire were also excluded. 

### 2.2. Baseline Assessment of Patients

Social, demographic, and baseline liver-disease-related data were collected at recruitment. Previous decompensation was defined according to Baveno VII (variceal bleeding, ascites, spontaneous bacterial peritonitis, and/or liver encephalopathy). The etiologies of the liver diseases considered were HCV, HBV, alcohol, autoimmune, MAFLD, or idiopathic. HCV and HBV serology were characterized in the Clinical Microbiology Department of the University Hospital Miguel Servet (Zaragoza, Spain) using cobas HCV viral load, cobas HCV genotyping tests, and cobas HBV viral tests (Roche, Basel, Switzerland). Patients were classified as having NAFLD if they met all the following criteria: (1) clinical diagnosis of MAFLD or CAP ≥ 263 dB/m, (2) exclusion of other liver diseases, and (3) lack of significant alcohol consumption of >21 standard drink per week in men and >14 standard drinks per week in women. Patients were classified as alcohol-related liver disease category if they fulfilled any of the following options: (a) clinical history of alcohol-related liver disease, or (b) a current significant alcohol consumption. Autoimmune hepatitis diagnosis was based on a combination of histological, serological, and exclusion criteria according to current European Association for the Study of the Liver (EASL) guidelines. Cirrhosis was defined as idiopathic in the absence of any other etiology after appropriate investigations. Duration of liver cirrhosis (since diagnosis) was also collected. Liver function was assessed using the Child–Pugh and MELD indices. 

The degree of comorbidity was evaluated using the Charlson index [17]. A specialist in endocrinology and nutrition performed nutritional and sarcopenia diagnoses. 

The diagnosis of malnutrition was performed according to the GLIM [18] criteria, classified as mild or severe malnutrition according to the cut-off points described in these criteria. These consensus criteria among the leading scientific nutrition societies require the fulfillment of at least one phenotypic criterion (unintentional weight loss, low BMI, and/or low muscle mass) and at least one etiological criterion (reduced nutrient intake or assimilation and/or presence of inflammation). To meet the inflammation criterion, any of the following assumptions were considered: a C-reactive protein value above 5 mg/dL, and/or concomitant acute inflammatory/infectious pathology. 

The diagnosis of sarcopenia was determined using the EWGSOP2 [19] criteria that recommend four steps: (1) use of the SARC-F [20] scale as screening for clinical suspicion, (2) muscle strength by hand grip strength (HGS) for probable sarcopenia, (3) muscle quantity by bioimpedance for confirmed sarcopenia, and (4) physical performance by gait speed (GS) for severe sarcopenia. 

HGS was measured with a Jamar model 5030J1 hand-held hydraulic dynamometer (Sammons Preston Inc., Bolingbrook, IL, USA). Participants squeezed the dynamometer with maximal isometric effort. The measurement was performed three times following Roberts’ protocol [21]. This protocol has evidence good to excellent (r > 0.80) test–retest reproducibility and excellent (r = 0.98) inter-rater reliability [22]. HGS results were classified according to EWGSOP2 as probable sarcopenia if HSG < 27 kg for men and <16 kg for women [23].

Muscle mass (kg/m^2^) was measured by bioimpedance (BIA) (Akern BIA 101 SMT device, Florence, Italy). Electrical parameters obtained with BIA were converted to appendicular skeletal muscle mass (ASM) using the validated Sergi equation [24]. As recommended by EWGSOP2, the sarcopenia cut-off points for ASM/height2 (ASMI) used were <7.0 kg/m^2^ for men and <5.5 kg/m^2^ for women [25].

The LDUST was administered under the supervision of one of the investigators, who provided assistance if needed. The LDUST consists of 6 questions with three possible options each. These questions focus on various aspects related to dietary intake (item 1), weight loss (item 2), fat loss (item 3), muscle loss (item 4), fluid retention (item 5), and patient’s ability to perform daily activities (item 6). The choice of 2 or more options B or C made the test pathological, and it was considered that there was a risk of malnutrition.

Patients were categorized into the exposed cohort if they scored “at risk” on the LDUST questionnaire and into the unexposed cohort if they scored “normal” on the LDUST questionnaire.

### 2.3. Follow-Up

Follow-up was carried out in outpatient consultations at least every six months, adapting the interval to the clinical criteria of the hepatologist responsible for the patient. Decompensations during follow-up, as well as hospital admissions, emergency cares, and death, were recorded. 

The need to increase the dose of diuretics or to place a TIPS, as well as the occurrence of severe infections, were also recorded. Severe infection was that which required intravenous treatment, drainage, and/or surgery for its treatment.

### 2.4. Ethical Statement

The study was conducted in accordance with the Declaration of Helsinki, and this study was evaluated and approved by the Clinical Research Ethics Committee of Aragon (CEICA). The code’s study was PI19/178. Informed consent was collected from all patients who agreed to participate in the study.

### 2.5. Statistical Analysis

For the description of the variables, percentages were used for qualitative variables and mean and standard deviation for quantitative variables. Fisher’s exact test was used to evaluate the association between two qualitative variables, while Studen’s *t*-test or the Mann–Whitney U-test were used to test for differences among normally and non-normally distributed variables, respectively. The evaluation of normal distribution relied on the Kolmogorov–Smirnov test, ensuring the appropriateness of chosen statistical tests based on the underlying data distribution.

Survival analysis was carried out using a multivariable Cox regression model. Right censored models accounted for cases where the event of interest had not yet occurred by the end of the follow-up, ensuring that these cases contributed to the analysis. Hazard ratios (HRs) represent the relative likelihood of an event (time to hospital admission, death, decompensation, and need for emergency care) occurring in one group compared to another in survival analysis. A HR greater than 1 indicates a higher hazard or risk of the event, while a HR less than 1 signifies a lower risk. The covariates assessed in the cox-regression model were sex, age, Child, MELD, sarcopenia, diagnosis of malnutrition, and previous decompensation. Sensitivity–specificity ROC curves were used to compare the predictive ability of the different tools, and differences were evaluated using the DeLong test [26].

Logistic regression models were used to assess the predictive ability of a combined Child/LDUST and MELD/LDUST model. The decompensation variable was used as the dependent variable. First, the combination of the variables was tested to improve the predictive ability of the model using the Akaike information criterion (AIC). This criterion was employed in our logistic regression model to facilitate the selection of the most suitable predictive model for combined variables. By striking a balance between model fit and complexity, AIC aids in preventing overfitting and guides the inclusion of relevant variables while penalizing unnecessary complexity. Subsequently, the coefficients of this model were used to calculate the new Child-LDUST and MELD-LDUST variables. Finally, the ROC curves of the original Child/MELD variable were compared with those of the new Child-LDUST and MELD-LDUST variables, respectively, using the DeLong test [23]. 

The statistical analysis was conducted using the user-friendly and open-source Jamovi software (Version 2.3.16, retrieved from https://www.jamovi.org, (accessed on 27 Augst 2023). The significance level for all tests was set at *p* < 0.05. 

## 3. Results

### 3.1. Baseline Patient Characteristics

**Figure 1:**
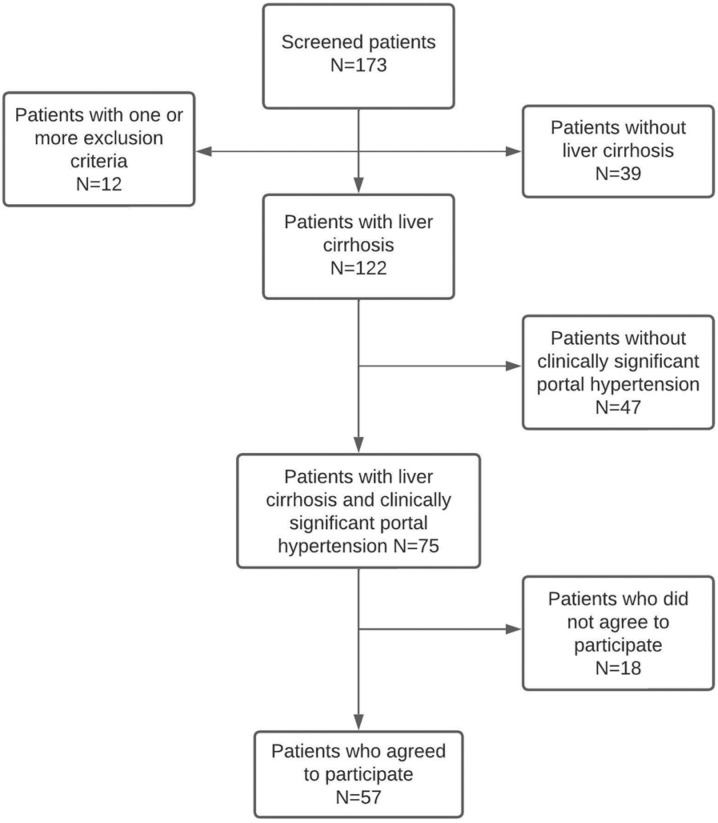
Patient’s flowchart.

A total of 57 patients (36.8% female, mean age 63.5 ± 9.9 years) were enrolled. The patient’s flowchart is shown in Figure 1. The most frequent etiology of liver cirrhosis was alcohol (50.9%), followed by chronic HCV infection (22.8%). Only 7% of patients had active alcohol consumption at the time of assessment. All patients with hepatitis C had previously received treatment with direct-acting antivirals with a sustained viral response. All HBV patients were on treatment with nucleoside analogs (entecavir or tenofovir).

Regarding liver function at inclusion, more than half of the patients had a Child grade A (56.1%), 38.6% grade B, and 5.3% grade C. A total of 80.7% had decompensated cirrhosis, i.e., had at least one previous decompensation, the most frequent being ascitic decompensation (57.9%).

Of the 57 patients evaluated, 31 (54.40%) were on malnutrition risk according to the LDUST.

Clinical and demographic variables did not show statistically significant differences according to the LDUST questionnaire score. All results can be seen in Table 1. 

The prevalence of malnutrition according to GLIM criteria was 39.3% (16.1% mild and 23.2% severe). Regarding the assessment of sarcopenia, 14.3% of the patients had sarcopenia. As can be seen in Table 2, patients with a risk LDUST had a higher prevalence of sarcopenia and malnutrition according to GLIM than patients with a normal LDUST.

### 3.2. Results during Follow-Up 

Patients were followed up for 3.5 years or until death. The median follow-up was 41 [6.9–44.2] months. There were no losses during follow-up. 

In the univariate analysis, undernourished patients according to the LDUST presented elevated mortality and increased episodes of hospital admission, decompensation, emergency department visits, ascites, need for increased dose of diuretics, evacuating paracentesis, hepatic encephalopathy, and severe infection (Table 3). Although not statistically significant, a similar trend was observed in the occurrence of gastrointestinal bleeding.

**Figure 2:**
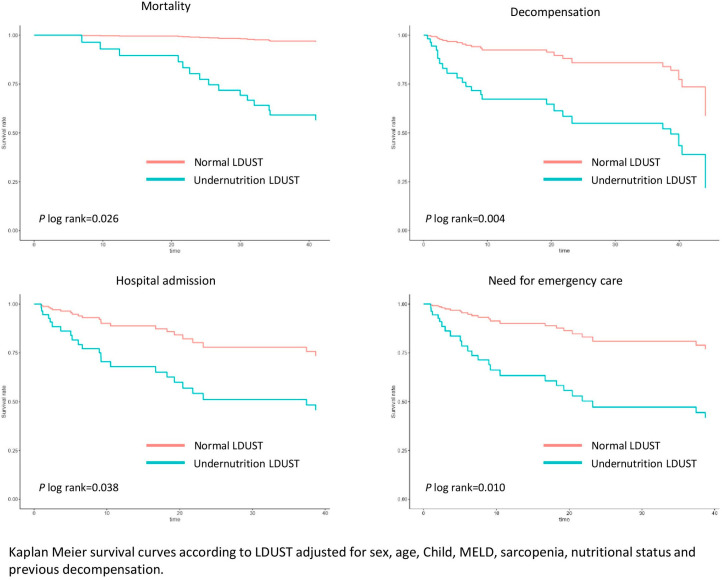
Adjusted survival curves for LDUST questionnaire score.

The survival analysis was adjusted for sex, age, comorbidity, presence of previous decompensation, sarcopenia, malnutrition according to GLIM criteria, and liver function of the patients. Compared to well-nourished patients, those affected with undernutrition according to LDUST had an increased risk of mortality (HR 14.72 (95% CI 1.93–112.13), *p* = 0.001), decompensation (HR 10.16 (95% CI 3.02–34.17), *p* = 0.001), admission (HR 9.39 (95% CI 2.77–31.87), *p* = 0.007), and emergency care (HR 9.93 (95% CI 2.94–33.54), *p* = 0.009) during follow-up. These analyses are shown in Figure 2.

Analyzing only Child A patients, those with undernutrition were also at higher risk of mortality (23.5% vs. 0%, *p* = 0.019), admission (HR 10.28 (95% CI 1.23–85.96), *p* = 0.001), ED care (10.28 (95% CI 1.23–85.96), *p* = 0.001), and decompensation (HR 8.79 (95% CI 1.04–74.24), *p* = 0.004). Significance was also reached in this analysis for the number of admissions (0.8 ± 1.4 vs. 0.0 ± 0.2, *p* = 0.025), the occurrence of ascites (29.4% vs. 0%, *p* = 0.008), the need for diuretic increase (29.4% vs. 0%, *p* = 0.008), and need for evacuative paracentesis (29.4% vs. 0%, *p* = 0.008).

### 3.3. Comparison of Predictive Ability

Given the observed results, the predictive capacity of the two main models of liver function (Child–Pugh and MELD) was compared with the LDUST tool for predicting death and the appearance of cirrhosis decompensation. 

**Figure 3:**
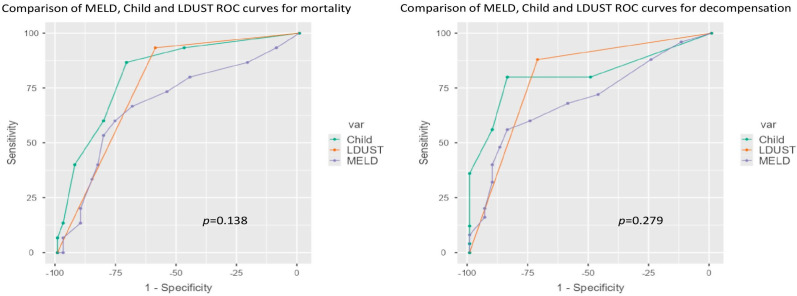
ROC curve comparison according to mortality and decompensation.

In both cases, no significant differences were observed between the area under the ROC curve (AUC-ROC) of the three tools for either death (LDUST 0.764, MELD 0.683, Child 0.819, *p* = 0.138) or occurrence of decompensation (LDUST 0.799, MELD 0.700, Child 0.811, *p* = 0.279) (Figure 3). 

Finally, the usefulness of the LDUST to improve the predictive ability of the Child–Pugh and the MELD scores was evaluated. Adding the LDUST score increased their predictive ability in both scales. Accordingly, new Child-LDUST and MELD-LDUST indices were created using the coefficients extracted from these models: Child-LDUST = 1.3 × Child points + 4.1 (for at-risk LDUST status); MELD-LDUST = 0.22 × MELD points + 2.8 (for at-risk LDUST status) (Table 4). 

**Figure 4:**
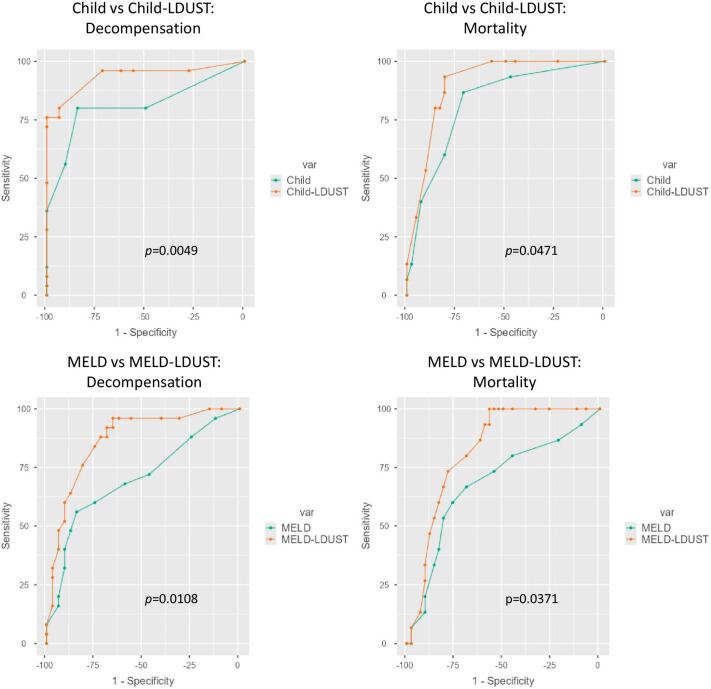
Comparative ROC curves between the original Child and MELD models and the Child-LDUST and MELD-LDUST combinations.

ROC analyses of these variables were then made and compared with the ROC curve of the original ones. Compared to the original Child and MELD scores, their combination with LDUST improved the AUC to predict decompensation (0.936 Child-LDUST vs. 0.811 Child; (*p* = 0.005)) and (0.904 MELD-LDUST vs. 0.819 MELD; (*p* = 0.011)), and mortality (0.866 LDUST-Child vs. 0.700 Child; (*p* = 0.047)) and (0.824 MELD-LDUST vs. 0.683 MELD; (*p* = 0.037)).The differences were statistically significant in both cases (Figure 4). 

The values of the respective AUC-ROC curves and the net differences are shown in Table 5.

## 4. Discussion

Our study is the first one to demonstrate in a prospective cohort the ability of the LDUST to predict the outcome of patients with cirrhosis, including some of the most clinically relevant events, such as death, new or further decompensation, and hospital admission. Moreover, combining the LDUST with the MELD and Child scores significantly improves their predictive ability.

Given the chronic nature of numerous medical conditions, a substantial proportion of our followed patients currently maintain stable health statuses. Consequently, the need for tools that can predict the risk of decompensation is growing in significance. These tools must possess attributes of simplicity and rapidity, ensuring their practical applicability in clinical settings. The paradigm of these tools is the ECOG scale [27,28] used mainly in oncology. Relying solely on the patient’s overall condition, this scale has demonstrated its efficacy in foreseeing clinical trajectories, treatment responses, and the onset of adverse events.

In the case of cirrhosis, the most used indices to predict progression are the Child–Pugh and MELD scores. The latter, helpful for prioritization on the transplant list, uses only analytical variables. The Child–Pugh score also considers the presence of ascites and hepatic encephalopathy. However, both have limitations. The first is the use of a parameter such as the INR, which can be altered for many reasons other than cirrhosis and cannot be directly assessed in patients on anticoagulant treatment with warfarin or acenocoumarol [29,30,31]. Moreover, both indices have little predictive capacity in the early stages of liver cirrhosis, especially when there has not yet been any decompensation [32,33,34]. Therefore, the search for and development of new models has been a constant in hepatology research [35,36,37], although almost always combining the parameters already used in these indices (bilirubin, creatinine, albumin, platelets, INR) with other analytical values or invasive tests, such as measurement of the portal pressure gradient. Prospective studies (reviewed by D’amico et al. [32]) showed that the most frequently associated variable with mortality was the Child–Pugh index (as well as its individual components) and age. Apart from the latter, only the MELD score, portal pressure gradient, and the presence of hepatocarcinoma were of note as predictors.

Recently, other concomitant conditions such as frailty, malnutrition, comorbidity, or minimal hepatic encephalopathy have gained importance [38,39,40]. These conditions, which are highly prevalent in patients with cirrhosis, significantly impact the course of the disease. However, their measurement is complex, even more so in patients with cirrhosis due to disease-specific alterations such as fluid retention. 

In our sample, the prevalence of sarcopenia and malnutrition reached, respectively, 14.3% and almost 40% according to the GLIM criteria. Although both conditions are associated with patient survival, their assessment is complex and time-consuming. It usually requires the collaboration of an endocrinologist and nutritionist, which makes their widespread application in clinical practice difficult. 

The LDUST was initially developed as a malnutrition screening questionnaire for liver cirrhosis patients. This need arose because ascites and/or edema, and their subsequent weight gain, posed a problem compared to conventional screening tests (MUST, MST, or MNA-SF) [12,41]. Studies in outpatient cohorts have shown its usefulness as a screening test [12,13,42]. Accordingly, the European clinical practice guideline on nutrition in patients with liver disease [14] suggests using this tool in outpatients. LDUST it is a self-administered questionnaire based on the patient’s subjective perception, so it follows a similar mechanism to a patient-reported outcome measure (PROM), providing an added dimension to the conventional way of assessing the symptoms and impact of a disease. While in other pathologies, such as inflammatory bowel disease, PROMs are more developed and widely used, this is not yet the case in liver cirrhosis [43].

Our results indicate that patients diagnosed as malnourished according to the LDUST had a higher prevalence of sarcopenia (23.3% vs. 3.8%) and malnutrition (60% vs. 15.4%) according to GLIM compared to their non-malnourished counterparts. Similarly, undernourished patients trended towards worse liver function (58.1% Child B/C) vs. 24.9% Child B/C in patients with normal LDUST. This indicates that the LDUST goes beyond assessing nutritional status and selects patients with severe features, which partly explains its ability to predict subsequent clinical outcomes. 

Within our cohort, individuals displaying an “at-risk” LDUST score distinctly exhibited less favorable outcomes throughout the follow-up period compared to those with a normal LDUST score. Our survival analysis unequivocally demonstrates that undernourished patients faced diminished overall survival, along with reduced durations of decompensation-free, admission-free, and emergency-department-free survival. Remarkably, the association between LDUST scores and adverse outcomes remained significant, even after accounting for variables such as liver function, malnutrition, sarcopenia, and comorbidity. This independent relationship underscores the robustness and clinical relevance of the LDUST result. In terms of other events, the ability of the LDUST questionnaire is noteworthy in terms of predicting the occurrence of complications related to ascitic decompensation, including increased diuretic dosage and the need for evacuative paracentesis. This is likely explained because the questionnaire directly assesses the patient’s perception of possible fluid retention. 

The results observed in the subgroup of patients with good liver function (Child A) are especially worth noting. An altered LDUST questionnaire was associated with a lower overall, admission-free, and decompensation-free survival in these patients at low risk of decompensation. In our work, no Child A patient with a normal LDUST died during follow-up, compared to 23.5% of those with risk LDUST. This translates into a negative predictive value for the test of 100% in this subgroup. Similarly, patients with a risk LDUST had a higher risk of edematous-ascitic decompensation during follow-up. In clinical practice this translates into that patients with a normal LDUST would have a very low risk of decompensation and mortality, making it possible to tailor the follow-up and reduce the need for controls, examinations, and analyses. It should be borne in mind that this subgroup of patients with non-compromised liver function is the most in need of malnutrition predictive tools. As seen in the systematic review by d’Amico [30], in the studies on compensated patients, the variables with the capacity to predict mortality were much fewer, and some, such as the MELD index, were not useful in this group. Furthermore, this emphasize that, although the LDUST score is associated with the results of liver function indices, it can discriminate patients at higher risk of poor outcomes independently of liver function.

Since LDUST, MELD, and Child indices use different parameters, we wanted to test whether their combination may improve their individual predictive ability. In our population, combining either MELD or CHILD function indices with the LDUST tool significantly improved in a clinically relevant way the ability to predict both liver decompensation and mortality. The difference in AUC was 0.141 and 0.166 with respect to the isolated MELD index for mortality and decompensation and 0.085 and 0.125 concerning the Child index. The improvement observed by linking the LDUST to the Child index was smaller than that obtained by linking it to the MELD index. This is probably because the Child index has better predictive capabilities than the MELD index in compensated patients, so the potential gain is smaller. Nevertheless, the improvement is equally substantial. Although these results need to be validated in an external population, they would be of great clinical relevance. Recently, Orman et al. reported the use of PROMs combined with liver function indices to predict events in cirrhotic patients [44]. However, their results showed a very discrete improvement in the predictive ability of hospital readmission. In our opinion, the main limitation of the work of Orman et al. is the use of tools for assessing PROs that are not specific to cirrhosis patients. The use of tools specifically designed for patients with liver cirrhosis may hence be key to achieve good results. Numerous studies have shown that it is essential to use tools specific to the patient’s specific pathology, as has been seen in several chronic pathologies [45,46,47].

The main strengths of our study are its prospective nature and the long follow-up time. These characteristics have allowed us to carry out an exhaustive characterization of the population, including liver function, nutritional status, sarcopenia, and comorbidity, thus avoiding possible confounding factors in the analysis. Furthermore, the population is representative of the population usually seen in hepatology consultations, which increases the external validity of our study. Some limitations also warrant consideration. The main limitation includes the relatively modest sample size and single-center nature of the study, suggesting a need for validation via larger multi-center studies. Additionally, potential selection bias due to the study’s design and selection based on specific criteria, as well as the inherent risk of unmeasured variables influencing outcomes, merit acknowledgment as potentially may lead to a lack of generalizability. Lastly, while data quality could be affected by recall or social desirability bias in patient-reported outcomes, these limitations were addressed through rigorous methodology and sensitivity analyses. 

The practical implications stemming from these findings hold significant clinical relevance. As delineated earlier, an array of tools exists for nutritional assessment in liver cirrhosis patients. Nonetheless, our results underscore the profound prognostic value inherent in the LDUST tool. Consequently, patients exhibiting at-risk scores, particularly those grappling with compromised liver function, should be promptly referred to specialists in endocrinology and nutrition for thorough evaluation. Notably, the LDUST’s capacity to identify patients who might evade detection through alternative means, such as the Child A subgroup, facilitates timely intervention. This preemptive approach serves to avert complications, diminish morbidity, and ultimately reduce mortality risks, illustrating the pivotal role of LDUST in optimizing patient outcomes.

The LDUST is a simple and easy-to-administer questionnaire that goes beyond being a malnutrition screening tool and can predict the risk of death and complications in outpatients with liver cirrhosis. Its predictive capacity is comparable to that of liver function models, and its combination with these models significantly improves their predictive capacity. We envision future research directions that could build on these findings, such as exploring the LDUST’s applicability in different liver diseases or assessing its utility in predicting response to specific treatments.

## Figures and Tables

**Table 1:** Baseline characteristics related to liver cirrhosis as a function of the LDUST.

	No Undernutrition (n = 26)	Undernutrition (n = 31)	All (N = 57)	*p*-Value
**Sex**				0.182
Female	12 (46.2%)	9 (29.0%)	21 (36.8%)	
Male	14 (53.8%)	22 (71.0%)	36 (63.2%)	
**Age (years)**				0.103
Mean (SD)	61.2 (10.3)	65.5 (9.2)	63.5 (9.9)	
**Alcohol**				0.221
No	23 (88.5%)	30 (96.8%)	53 (93.0%)	
Yes	3 (11.5%)	1 (3.2%)	4 (7.0%)	
**Etiology**				0.497
HCV	6 (23.1%)	7 (22.6%)	13 (22.8%)	
HBV	3 (11.5%)	1 (3.2%)	4 (7.0%)	
Alcohol	14 (53.8%)	15 (48.4%)	29 (50.9%)	
Autoimmune	0 (0.0%)	3 (9.7%)	3 (5.3%)	
MAFLD	1 (3.8%)	1 (3.2%)	2 (3.5%)	
Idiopathic	2 (7.7%)	4 (12.9%)	6 (10.5%)	
**Child–Pugh**				0.090
A	19 (73.1%)	13 (41.9%)	32 (56.1%)	
B/C	7 (26.9%)	18 (58.1%)	25 (43.9%)	
**MELD**	9.7 (3.0)	11.4 (4.1)	10.6 (3.7)	0.096
**Charlson Index**				0.895
Mean (SD)	4.2 (1.4)	4.3 (1.9)	4.3 (1.7)	
**Previous decompensation**				0.508
No	6 (23.1%)	5 (16.1%)	11 (19.3%)	
Yes	20 (76.9%)	26 (83.9%)	46 (80.7%)	
**Ascitis**				0.571
No	12 (46.2%)	12 (38.7%)	24 (42.1%)	
Yes	14 (53.8%)	19 (61.3%)	33 (57.9%)	
**Esophageal varices**				0.176
No	5 (19.2%)	5 (16.1%)	10 (17.5%)	
Small	18 (69.2%)	16 (51.6%)	34 (59.6%)	
Large	3 (11.5%)	10 (32.3%)	13 (22.8%)	

HCV: hepatitis C virus; HBV: hepatitis B virus; MAFLD: metabolic-associated fatty liver disease; MELD: Model of End Stage Liver Disease.

**Table 2:** Baseline nutritional characteristics according to the result of the LDUST.

	No Undernutrition (n = 26)	Undernutrition (n = 31)	All (N = 57)	*p*-Value
**GLIM**				0.001
Normal	22 (84.6%)	12 (40%)	34 (60.7%)	
Mild malnutrition	3 (11.5%)	6 (20%)	9 (16.1%)	
Severe malnutrition	1 (3.8%)	12 (40%)	13 (23.2%)	
**Sarcopenia**				0.038
Normal	25 (96.2%)	23 (76.7%)	48 (85.7%)	
Sarcopenia	1 (3.8%)	7 (23.3%)	8 (14.3%)	
**Abdominal perimeter**				0.642
Normal	8 (30.8%)	11 (36.7%)	19 (33.9%)	
Metabolic syndrome criteria *	18 (69.2%)	19 (63.3%)	37 (66.1%)	
**Calf circumference**				0.038
Undernourished	1 (3.8%)	7 (23.3%)	8 (14.3%)	
Normal	25 (96.2%)	23 (76.7%)	48 (85.7%)	
**Arm circumference**				0.029
Undernourished	0 (0%)	6 (20%)	6 (10.7%)	
Regular	1 (3.8%)	3 (10%)	4 (7.1%)	
Normal	25 (96.2%)	21 (70%)	46 (82.1%)	
**Strength (hand-grip)**				0.122
Normal	22 (84.6%)	20 (66.7%)	42 (75%)	
Low	4 (15.4%)	10 (33.3%)	14 (25%)	
**BMI (kg/m^2^)**				0.346
<19	0 (0%)	2 (6.7%)	2 (3.6%)	
19–21	2 (7.7%)	3 (10%)	5 (8.9%)	
21–23	2 (7.7%)	5 (16.7%)	7 (12.5%)	
>23	22 (84.6%)	20 (66.7%)	42 (75%)	
**FFMI**				0.097
Normal	26 (100%)	27 (90%)	53 (94.6%)	
Low	0 (%)	3 (10%)	3 (5.4%)	

GLIM: Global Leadership Initiative on Malnutrition; BMI: body mass index; FFMI: free fat mass index; * (>102 cm in men and >88 cm in women).

**Table 3:** Events during follow-up based on LDUST test results.

	No Undernutrition (n = 26)	Undernutrition (n = 31)	All (N = 57)	*p*-Value
**Exitus**				<0.001
No	25 (96.2%)	17 (54.8%)	42 (73.7%)	
Yes	1 (3.8%)	14 (45.2%)	15 (26.3%)	
**Emergency care**				<0.001
No	23 (88.5%)	10 (32.3%)	33 (57.9%)	
Yes	3 (11.5%)	21 (67.7%)	24 (42.1%)	
**Hospital admission**				<0.001
No	23 (88.5%)	11 (35.5%)	34 (59.6%)	
Yes	3 (11.5%)	20 (64.5%)	23 (40.4%)	
**Number of admissions**				<0.001
Mean (SD)	0.2 (0.8)	1.9 (2.0)	1.1 (1.8)	
**ICU admission**				0.103
No	26 (100.0%)	28 (90.3%)	54 (94.7%)	
Yes	0 (0.0%)	3 (9.7%)	3 (5.3%)	
**New or further decompensation**				<0.001
No	23 (88.5%)	9 (29.0%)	32 (56.1%)	
Yes	3 (11.5%)	22 (71.0%)	25 (43.9%)	
**Ascitis (new or worsen)**				<0.001
No	26 (100.0%)	15 (48.4%)	41 (71.9%)	
Yes	0 (0.0%)	16 (51.6%)	16 (28.1%)	
**Increased doses of diuretics**				<0.001
No	26 (100.0%)	16 (51.6%)	42 (73.7%)	
Yes	0 (0.0%)	15 (48.4%)	15 (26.3%)	
**Evacuative paracentesis**				<0.001
No	26 (100.0%)	17 (54.8%)	43 (75.4%)	
Yes	0 (0.0%)	14 (45.2%)	14 (24.6%)	
**Variceal bleeding**				0.076
No	25 (96.2%)	25 (80.6%)	50 (87.7%)	
Yes	1 (3.8%)	6 (19.4%)	7 (12.3%)	
**Encephalopathy**				0.042
No	24 (92.3%)	22 (71.0%)	46 (80.7%)	
Yes	2 (7.7%)	9 (29.0%)	11 (19.3%)	
**Severe infection**				0.042
No	24 (92.3%)	22 (71.0%)	46 (80.7%)	
Yes	2 (7.7%)	9 (29.0%)	11 (19.3%)	

ICU: intensive care unit.

**Table 4:** Regression models and coefficients.

Coefficients of the Child-LDUST Model—Decompensation				
**Predictor**	**Exp (B)**	**EE**	**Z**	***p***
Constant	−11.18	3.001	−3.72	<0.001
LDUST				
Risky–normal	4.10	1.207	3.40	<0.001
Child (points)	1.29	0.379	3.41	<0.001
Coefficients of the MELD-LDUST model—Decompensation				
**Predictor**	**Exp (B)**	**EE**	**Z**	***p***
Constant	−4.475	1.298	−3.45	<0.001
LDUST				
Risky–normal	2.841	0.750	3.79	3.79
Child (points)	0.227	0.101	2.24	2.24

Estimators represent log odds of “decompensation = yes” vs. “decompensation = no” when quantitative variables take the value 0 and categorical variables the reference value.

**Table 5:** Values of areas under the curve and net differences between the indices and the corresponding combined model.

	Decompensation		Mortality	
	AUC Value	Difference	AUC Value	Difference
Child	0.811	0.125	0.700	0.166
Child-LDUST	0.936		0.866	
MELD	0.819	0.085	0.683	0.141
MELD-LDUST	0.904		0.824	

## Data Availability

The data presented in this study are available on request to the corresponding author with prior authorization of our Ethical Committee that can be obtained at https://www.iacs.es/investigacion/comite-de-etica-de-la-investigacion-de-aragon-ceica/ceicaevaluaciones-y-otras-presentaciones (accessed on 25 July 2023).
